# COVID-19 Vaccinations: A Comprehensive Review of Their Safety and Efficacy in Special Populations

**DOI:** 10.3390/vaccines9101097

**Published:** 2021-09-28

**Authors:** Zhipeng Yan, Ming Yang, Ching-Lung Lai

**Affiliations:** 1 Department of Medicine, Queen Mary Hospital, The University of Hong Kong, Hong Kong, China; 2 Department of Ophthalmology, Li Ka Shing Faculty of Medicine, The University of Hong Kong, Hong Kong, China; hrmeym@hku.hk

**Keywords:** COVID-19 vaccinations, pregnancy, frailty, comorbidities

## Abstract

**COVID-19** has been spreading worldwide since late 2019. There is no definitive cure to date. Global vaccination programs are urgently required to confer herd immunity, reducing the incidence of COVID-19 infections and associated morbidity and mortality. However, a significant proportion of special populations are hesitant to receive vaccination due to their special conditions, namely, age (pediatrics and geriatrics), immunocompromised state, autoimmune diseases, chronic cardiovascular and pulmonary conditions, active or treated cancers, and pregnancy. This review aims to evaluate the existing evidence of COVID-19 vaccinations on these special populations and to provide clues to guide vaccination decision making to balance the benefits and risks of vaccinations.

## 1. Introduction

COVID-19 has been spreading globally since late 2019 [1]. There is no definitive cure to date. The consequences of being infected with COVID-19 can be multi-faceted, such as multisystem inflammatory syndrome in children, acute multi-organ failure in adults, and long COVID-19 syndrome in all recoverees [2,3,4,5]. Global vaccination programs and multiple preventive measures are the most effective ways to curb rapid transmission. The progress of clinical trials has been accelerating globally to test the protective efficacy of different vaccines [6]. However, there remains a great hesitancy to receive the vaccines due to various reasons, such as fear of the side effects of the vaccination, especially in subjects with underlying co-morbidities [7]. The hesitancy rate of vaccination was found to be 19% in the United States [8], 35% in Ireland, and 31% in the United Kingdom [9]. The major factor is the uncertainty of the safety and efficacy of vaccination in high-risk groups. This review aims to review the most updated evidence concerning COVID-19 vaccination in high-risk groups to facilitate their decision-making.

## 2. Focus of Attention: To Receive Vaccination or Not?

COVID-19 infection is associated with many complications, both short-term and long-term. These include long COVID-19 syndrome in COVID-19 survivors, multisystem inflammatory syndrome in the elderly, and myocarditis in pediatric populations [10,11]. This will create extra problems for the future healthcare system in terms of medical follow-up, rehabilitation, and financial burden [4].

Vaccination confers herd immunity through induction of memory immune responses to activate immunological defense against SARS-CoV-2 infections [12,13,14,15,16]. However, there is still a potential risk of illness after successful vaccination and subsequent infection with variant strains [17]. In view of this, some vaccine trials have demonstrated their reduced protective efficacy to variant strains of SARS-CoV-2 infections [13,14,15,18,19]. It is therefore vital to provide continuous supports to prevent infection by high compliance of global vaccination, social distancing, wearing of masks, and personal hygiene.

The vaccination response usually requires the introduction of foreign antigens or signaling such as mRNA, inactivated virus, or non-replicating viral vector; protein subunits; or live attenuated virus to trigger immune responses, as shown in experimental clinical trials [6]. This is not without risk, since some serious adverse events have been reported, especially in subjects with risk factors such as extremity of age; being immunocompromised; having autoimmune diseases, chronic respiratory conditions, chronic cardiovascular conditions, or malignancy; or being pregnant. Vaccination is a viable way to reduce COVID-19 hospital admissions and severity of disease manifestations by attenuation of viral RNA load, risk of febrile symptoms, and duration of illness [12]. Therefore, global governments have advocated various incentives for boosting the vaccination rates.

Meanwhile, there has been serious adverse outcomes observed such as stroke, coagulation and platelet disorders, myocarditis, and other disorders [20,21,22,23]. This deters individuals, especially high-risk groups, to receive vaccination. Currently, only limited evidence exists, and therefore it is uncertain as to whether high-risk groups should receive the vaccination. This review aims to look into the current evidence of vaccinations for nine types of special populations and provide clues to vaccination decision making.

### 2.1. Adolescents and Children

Early studies show that COVID-19 infections of adolescents and children were milder and with better prognosis compared with adults [24]. This may be associated with the innate immune system of children [25]. The immune preparedness of children to cope with SARS-CoV-2 infection has been closely related to the production of natural antibody (IgM) with broad reactogenicity, as well as the possible role of CD27-related memory B cells (MBC). The broad-reactogenic natural antibody aids the clearance of the virus and prevents reinfection, as evidenced in other coronavirus infections such as SARS [26]. The highly plastic immune system of children coordinated by CD-27 MBC shows resilience and rapidity in secretions of antigen-specific antibodies, protecting children from the infection [27]. In spite of this, children are still at risk for SARS-CoV-2 infection and severe disease manifestations [24,28]. They remain at low risk of COVID-19 mortality but have a high morbidity risk [29]. In a study including mortality of young people (less than 19 years old) in seven countries, the overall mortality rate of COVID-19 was 35.26 per 100,000 persons [29]. Multisystem inflammatory syndrome in children (MIS-C) has been reported in the literature. The incidence of MIS-C was 5.1 persons per million person-months, and 316 persons per million SARS-CoV-2 infections in persons younger than 21 years old [30]. Vaccinations may provide additional protections to their innate immune system; thus, vaccination should be encouraged if the vaccine is safe and efficacious [31].

A recent study shows that the use of BNT162b2 (developed by Pfizer) COVID-19 vaccine in adolescents has favorable safety outcomes and protective efficacy [32]. In a study involving 2260 adolescents 12 to 15 years of age receiving the Pfizer COVID-19 vaccination randomized in a 1:1 ratio to receiving vaccine or placebo, the observed vaccine efficacy was 100% (95% CI, 75.3 to 100). The side effects documented were transient mild-to-moderate reactogenicity: injection-site pain (79 to 86%), fatigue (60 to 66%), and headache (55 to 65%). There were no vaccine-related serious adverse events.

Many vaccine candidates have not published their clinical trials on adolescents and children. The detailed protective efficacy and adverse event profiles in individual vaccine designs are not clear at the time of writing; therefore, the vaccination rate among pediatric populations can only be estimated by hypothetic models. A global study involving over 17,000 pregnant women or mothers of children younger than 18 years old from 16 countries showed that the willingness to accept vaccinations was related to confidence in vaccine safety or effectiveness, worry about COVID-19, and belief in the importance of vaccines to their own country [33]. With 90% vaccine efficacy assumptions, 52.0% of pregnant women (*n* = 2747/5282) and 73.4% of mothers (*n* = 9214/12,562) indicated an acceptance of receiving the vaccination. A total of 69.2% of women (*n* = 11,800/17,054), both pregnant and non-pregnant, indicated an intention to vaccinate their children.

Various vaccination trials in pediatric populations have been designed. Pfizer started a staged enrolment of 2000 children aged 12–15 for their clinical trials, as described above [14]. The second phase will be in children aged 5–11 [34]. Moderna enrolled 3000 adolescents aged 12–17 with a randomization ratio of 1:1 to receive two shots of vaccine or placebo 4 weeks apart in December 2020 [35]. AstraZeneca enrolled 300 children aged 6–17 to receive either COVID-19 vaccines or control meningitis vaccine in February 2021 [36]. More studies are required to bridge the knowledge gap of safety and efficacy profiles of the use of COVID-19 vaccines in pediatric populations.

### 2.2. Geriatric Population

Geriatric populations are at high risk of COVID-19-associated mortality [37]. Their reduced immunity and age-related organ functional decline predispose them to a higher infection risk, leading to fatal outcomes [38,39]. Vaccination with BNT162b2 (developed by Pfizer) in Israel was shown to be successful in decreasing mortality risk and COVID-19-associated admissions in a nationwide vaccination program [40]. Elderly subjects therefore should be prioritized to receive vaccination if there are no contraindications, severe medical comorbidities, or frailty [41,42]. The first dose vaccination substantially reduced the risk of COVID-19-related hospitalizations in elderly, frail patients with co-morbidities [43], with the Pfizer vaccine BNT162b2 effectiveness being 71.4% (95% CI, 46.5–90.6) and ChAdOx1nCoV-19 (developed by Oxford University and AstraZeneca) 80.4% (95% CI, 36.4–94.5). However, the antibody responses may wane quickly, especially with the Pfizer vaccine. This requires earlier revaccination and/or an increased vaccine dose to ensure longer-lasting immunity [44].

ChAdOx1nCoV-19 (AZD1222) is an adenovirus-vectored vaccine developed by Oxford–AstraZeneca. It contains a full-length structural surface glycoprotein (spike protein) of SARS-CoV-2, with a tissue plasminogen activator leader sequence [45]. The phase II study of the ChadOx1 nCoV-19 vaccine in older adults (including 200 subjects aged over 70 years without severe comorbidity or frailty) showed that the vaccine was safe and well-tolerated, with immunogenicity similar to the younger groups [46]. This was consistent with the mRNA vaccine developed by Moderna, which showed a similar neutralizing antibody response in all patients independent of age. The phase I, dose-escalation, open-label trial of Moderna mRNA-1273 showed a strong immunogenic response and mild side effect profiles in older adults (at least 56 years old) [47]. In another study of Moderna vaccine involving 7000 patients over aged 65, and 5000 under 65 with high-risk chronic diseases (out of total study population of 30,000 participants), the trial showed 94.5% efficacy [48].

Contrary to the mRNA vaccine (developed by Moderna, the United States), the adenovirus-based vaccine (developed by Cansino, Tianjin, China) showed lower neutralizing antibody titers, lower T-cell-mediated immune responses, and lower incidence of adverse events in patients older than 55 years old, compared with the younger groups [49]. Another adenovirus-based vaccine, Ad26.COV2, developed by Johnson & Johnson, also showed a lower rate of adverse events and lower immunogenicity in older participants (older than 65 years, 36%) compared with younger people (younger than 65 years, 64%) [50].

A U.K. study comparing the vaccine effectiveness between BNT162b2 (Pfizer) and ChAdOx1-S (recombinant vaccine by AstraZeneca) in adults older than 70 years old showed that vaccinations with either BNT162b2 (Pfizer) or ChAdOx1-S (AstraZeneca) was associated with a significant reduction of symptomatic infections and severe COVID-19 admissions in older adults [51]. Protection duration was at least 6 weeks, while the second dose further increased protective efficacy against symptomatic disease, including protection against the B.1.1.7 variant. Overall, the combined analysis showed that the first and second dose vaccine effectiveness levels measured 14 days after the second dose were 70% (95% CI, 59–78) and 89% (95% CI, 85–93), respectively. Subgroup analysis of BNT162b2 (Pfizer) recipients showed a protective efficacy of 61% (95% CI, 51–69) 4 weeks after full-dose vaccinations, then plateaued thereafter. This was comparable with the result of ChAdOx1-S (AstraZeneca) recipients with protective efficacies at 4 and 6 weeks, being 60% (95% CI, 41–73) and 73% (95% CI, 27–90), respectively. The emergency hospital admission risk reductions were comparable in both vaccination groups: 43% (95% CI, 33–52) and 37% (95% CI, 3–59), respectively.

In terms of incidence of vaccine-associated adverse events, a recent study showed that the younger population (18–64 years old) has a higher incidence rate of adverse events compared to the older populations (65 years or older) [52]. However, a higher rate of *serious* adverse events is reported in older populations. The adjusted odd ratios (AOR) are: reporting serious adverse events AOR 6.26 (95% CI 5.00–7.84), death AOR 19.99 (95% CI 13.29–30.07), permanent disability AOR 2.08 (95% CI 1.07–4.04), and hospitalization AOR 2.96 (95% CI 2.28–3.85) in older populations, compared with the younger groups.

The COVID-19 mortality risk in unvaccinated geriatric populations is high [53,54,55,56,57]. For people between 50 and 65 years old with general good past health, the mortality risk is 0.5%. However, the mortality risk becomes 11.6% for patients in their mid-seventies [53]. Another study similarly shows that the mortality risk is 8.1 (95% CI 7.7–8.5) times higher in the elderly population (55–64 years old), compared with the younger population (younger than 55 years old) [54]. The mortality risk rises to 62 (95% CI 59.7–64.7) times higher in senior populations (older than 64 years old), compared with younger populations (55 years old) [54]. Aging (older than 75 years old) alone contributed to a fourfold increase of mortality risk in a British study of over 470,000 participants without additional risk factors [56].

The COVID-19 mortality risk is even higher in elderly unvaccinated inpatients with comorbidities [55]. The survival time from symptom onset is significantly shorter in elderly (>70 years old) versus young patients (<70 years old): median 29 versus 62 days (*p* < 0.01) [55]. The lethality rate of elderly patients (more than 60 years old) with one or more risk factors was 41.28% among hospitalized patients in a Brazilian study of more than 160,000 patients [57]. The lethality rate rises to 82.98% in these elderly patients with risk factors receiving invasive mechanical ventilation [57].

Thus, geriatric populations should consider their risk factors, risk of COVID-19-associated mortality, type of vaccination, and vaccination risks and benefits before making a vaccination decision. Counseling on individual risk profile should be done by healthcare workers if in doubt.

### 2.3. Immunocompromised Subjects

Immunocompromised subjects are more vulnerable to infections. This section includes transplant recipients, HIV-infection carriers, and advanced chronic liver diseases. Immunocompromised subjects have higher inpatient mortality risk compared with non-immunocompromised populations. The adjusted odd ratio (aOR) for in-patient death was 1.60 (95% CI 1.43–1.79) for overall immunocompromised patients [58]. The aOR of transplant recipients, solid organ neoplasm, and hematological neoplasm were 3.12 (95% CI 2.23–4.36), 1.39 (95% CI 1.18–1.63), and 2.31 (95% CI 1.76–3.03), respectively [58]. Compared with non-HIV carriers, the odds ratio of mortality in HIV-infection carriers is 1.52 (95% CI 1.25–1.85) in the United States [59]. Patients with liver cirrhosis complicated by COVID-19 have worse prognosis, compared with COVID-19 patients without liver cirrhosis (30% vs. 20%, *p* = 0.03) [60]. This is consistent with another study that showed that the 30-day mortality rate was 34% in patients with liver cirrhosis and COVID-19 infections [61]. Thus, immunocompromised subjects are at a high risk of mortality if complicated with COVID-19 infections. Vaccinations should be promoted in this group.

The data regarding the efficacy and safety profile of vaccination in solid organ transplant recipients are limited. The protective effect and duration of the vaccines are lower in solid organ transplant recipients, compared with general populations. A recent study of 436 solid organ transplant recipients showed that a low proportion of solid organ transplant recipients (17%) tested positive with SARS-CoV-2 antibody after their first dose of mRNA-vaccination (Moderna) [62]. Patients receiving anti-metabolite maintenance immunosuppression were less likely to develop antibody responses to vaccination, compared with recipients without immunosuppressive therapy (37% vs. 63%, respectively; adjusted incidence ratio IRR = 0.22, 95% CI 0.15–0.34; *p* = 0.002). Patients receiving mRNA-based vaccine mRNA-1273 (Moderna) were more likely to develop an antibody response than in BNT162b2 vaccine by Pfizer (69% vs. 31%, IRR = 2.15, 95% CI 1.29–3.57; *p* = 0.003) [62].

Another study with 658 solid organ transplant recipients also showed only 98 (15%) participants developed measurable antibody response after two doses of mRNA-based vaccinations (Moderna) [63]. Among 473 patients receiving anti-metabolites, 8% of them developed antibody response after two doses, compared with 32% of patients who developed antibody responses who did not receive anti-metabolite [63]. More research looking into alternative solutions is encouraged. These include adjustment of immunosuppressive regimens or delaying the vaccination schedule due to induction phase high-dose immunosuppression treatment. Recent evidence showed a third-dose mRNA vaccination (Pfizer) significantly improves the immunogenicity of the vaccine [64]. Meanwhile, barrier methods such as social distance and wearing of masks should still be advocated.

Transplant recipients have a higher risk of severe COVID-19 infections [65,66]; they are recommended to receive vaccinations, unless there are other contraindications. Their family members should also consider vaccination to prevent cross-infection between family members.

Patients with HIV infection (with CD4 counts less than 500 cells/µL) had a higher risk of hospitalization, intensive care unit admission, and/or death [67]. They may be reluctant to receive COVID-19 vaccinations due to various concerns. Most expressed concerns of safety about their health (*p* < 0.001), the requirement of mandatory vaccinations (*p* = 0.017), and their chronic disease status (*p* = 0.026). A COVID-19 vaccine hesitancy study of HIV patients showed that more than 50% of the participants expressed at least one COVID-19 misbelief or treatment hesitancy belief [68]. Further research on the safety and efficacy of COVID-19 vaccination in HIV patients, effective public education to remove vaccination misbeliefs, and campaigns to promote vaccination culture are required.

### 2.4. Autoimmune Diseases

Patients with autoimmune diseases require special care prior to vaccinations. Most early studies excluded patients with autoimmune diseases or immunocompromised states [69], although patients with moderate to high dose corticosteroid are at a high risk of COVID-19 infections [70,71]. Common agents used for autoimmune diseases include corticosteroid, rituximab, methotrexate, and other biologics. These may inhibit the humoral response of vaccinations, dampening the immune responses [72]. The adjusted odds ratios of inpatient mortality risk were 2.16 (95% CI 1.80–2.61), 1.97 (95% CI 1.33–2.91), and 2.06 (95% CI 1.64–2.60) for patients receiving systemic steroids, biologic treatments, and other immunosuppressants, respectively [58].

Previous experience of seasonal influenza vaccination showed that a 2-week discontinuation of methotrexate after vaccination did not cause an increase in rheumatoid arthritis disease activity, while rituximab retarded the immune responses of pneumococcal vaccinations [73,74]. Meanwhile, the American College of Rheumatology recommended withholding methotrexate, JAK-inhibitor, abatacept, and intravenous cyclophosphamide for 1 week after each vaccination dose rather than 2 weeks due to the increased risk of disease flare-up with longer discontinuation period [75]. Similar strategy of change of pharmacological regimen or temporary discontinuation of disease-modifying anti-rheumatoid agents can be considered to balance the treatment efficacy of autoimmune diseases and immunological efficacy of vaccinations [72,76]. However, no change of drugs regimen is required for patients taking hydroxychloroquine, leflunomide, sulfasalazine, mycophenolate mofetil, azathioprine, cyclophosphamide (oral), biologics (IL-1, IL-17, IL-12/IL-23, IL-23, IL-6R, TNF), belimumab, and oral calcineurin inhibitors [75].

Several recommendations are provided according to risk stratifications of patients and adjustment of timing for vaccinations [72]. Patients with anti-CD20 medications (e.g., rituximab) are recommended to vaccinate before the commencement of rituximab, or to avoid vaccinations 6 months post-rituximab [72]. This is because the previous experience of influenzae vaccinations showed that better humoral response was only observed more than 5 months after rituximab treatment [77]. The American College of Rheumatology recommends initiation of vaccination schedule about 4 weeks prior to the next dose of rituximab [45]. Patients should avoid vaccination during disease flare-up and taper steroid therapy to less than 10mg prednisolone daily [72]. Steroid dosage higher than 10 mg is associated with a greater degree of impairment of humoral immunity [78].

Vaccination may cause undesirable adverse events in patients with autoimmune diseases. It may cause acute disease flare-ups due to the molecular mimicry between the viral spike peptides and the patients’ self-peptides [79]. Another study showed the possibility of herpes virus reactivation after BNT162b2 (Pfizer) vaccinations in autoimmune rheumatoid arthritis patients [80]. Underlying mechanisms may be related to toll-like receptor activation by the vaccine, stimulating the induction of type I interferons and inflammatory cytokines that may contribute to reactivation of herpes zoster [81,82].

Vaccination should be done after balancing the benefit and risks under the supervision of healthcare workers with careful evaluations of age and co-morbidities. The American College of Rheumatology recommends all low-risk patients receive vaccinations as soon as possible. These include stable patients with low disease activity and patients with active but non-life-threatening diseases [75]. Patients with life-threatening diseases should defer their vaccination schedule until better disease control. Patients with rheumatological diseases should delay their second dose until they have recovered from the acute illness [83]. Patients should defer vaccinations for at least 90 days if they have received monoclonal antibody or convalescent plasma as part of the treatment of COVID-19 infections [84].

### 2.5. Cardiovascular Diseases

At the time of writing, there are no systematic studies investigating the safety and efficacy of COVID-19 vaccines for people with cardiovascular diseases. Some vaccine clinical trials have included patients with cardiovascular comorbidities.

Vaccination is encouraged in most people with cardiovascular diseases by the American Heart Association and the American College of Cardiology. This is because this group of people has a higher risk for severe diseases if infected [85,86], and the advantages of getting vaccinated outweigh the potential disadvantages. There are some reports on vaccine-associated myocarditis [22,87,88,89]. Further laboratory investigations showed elevated troponin with negative viral serologies, and cardiac magnetic resonance imaging (MRI) demonstrated edema and delayed gadolinium enhancement of the left ventricle in a midmyocardial and epicardial distribution [23]. This may be seen as a possible adverse reaction to warn against patients before vaccination [22,90].

If the disease symptoms such as chest pain and shortness of breath on exertion and hypertension are poorly controlled, deferred vaccination is recommended until the symptoms are controlled. For acute diseases, such as acute myocardial infarction, acute heart failure, and acute exacerbation of chronic heart failure, vaccination should be postponed until the patient is in a stable condition. With good drug control, patients should consult a doctor to determine whether or not to be vaccinated on the basis of the patient’s specific disease progression. The association between myocardial infarction (MI) and COVID-19 vaccination is unclear; MI were seen in some post-vaccination elderly without cardiovascular risk factors [91,92]. More search is required to establish the causal relationship between MI and COVID-vaccination to guide the decision making of post-MI patients.

There have been reports on post-vaccination myocarditis, pericarditis, vasculitis, and cardiac arrhythmia in patients with good general past health after mRNA vaccination for both Pfizer and Moderna [90,93,94]. The incidence rate of myocarditis or pericarditis is reported to be 12.6 cases per million doses of second-dose mRNA vaccine among individuals 12 to 39 years of age without a history of COVID-19 infections or comorbidities. These typically happen 2 to 3 days after the second dose of mRNA vaccination [94,95]. The median duration of myocarditis-related admission is 2 days (inter-quartile range of 2–3 days), with no readmissions or deaths [94]. With a median of 23.5 days of follow-up, the majority (65%) of patients experience symptoms resolution, and the remaining (35%) experience symptomatic improvements [94]. The immediate course of myocarditis and pericarditis following vaccination is generally mild and responsive to conservative treatments such as rest and treatment with anti-inflammatory drugs [96]. The association between chronic cardiovascular comorbidities and post-vaccination myocarditis remains uncertain to date. Further research is required to study their risk associations [96]. The vaccine-associated risk ratio of myocarditis is 3.24 (95% CI 1.55–12.44), while COVID-19 infection-associated myocarditis is 18.28 (95% CI 3.95–25.12) [97]. In view of the emerging evidence, the Advisory Committee on Immunization Practices of the United States recommends that the benefits of using mRNA vaccines under the Food and Drug Administration (FDA)’s emergency use authorization (EUA) outweighed the risks in all populations, including adolescents and young adults [98].

### 2.6. Chronic Respiratory Diseases

SARS-CoV-2 itself causes severe respiratory failure syndrome. A systematic review and meta-analysis analyzing over 690,000 patients with chronic lung conditions with COVID-19 infection showed that chronic lung conditions significantly increase the odds of poor clinical outcomes in patients with COVID-19: increased odds of hospitalization (odd ratio 4.23, 95% CI, 3.65–4.90), ICU admission (odd ratio 1.35, 95% CI 1.02–1.78), and mortality (odd ratio 2.47, 95% CI 2.18–2.79) [99]. Patients with chronic respiratory diseases (CRD) such as chronic obstructive pulmonary disease, bronchiectasis, cystic fibrosis, and interstitial lung disease have higher risks for severe COVID-19 hospital admissions and mortality risk [100]. The risk of COVID-19 patients developing ventilator-associated pneumonia and pulmonary embolism is higher than patients with influenza. The risk of both ICU and in-patient mortality was increased for patients with CRD, compared with COVID-19 patients without CRD: adjusted odd ratios were 1.34 (95% CI 1.28–1.41) and 1.19 (95% CI 1.14–1.25), respectively. Patients are recommended for vaccination [101]. However, no trials thus far have been performed specifically on patients with chronic respiratory conditions to date.

### 2.7. Diabetic and Centrally Obese Populations

Patients with diabetes and obesity have a high risk of severe diseases, intensive care unit admissions, and increased mortality with COVID-19 infections [102,103,104]. The possible underlying mechanisms may be related to immune dysfunction, increased susceptibility to inflammation, reduced viral clearance, and disturbed rennin–angiotensin–aldosterone system [105]. Good glycemic control does not provide significant improvement of clinical outcomes in diabetic patients with COVID-19 infections [106]. Thus, prevention of COVID-19 infections is the key form of management for diabetic patients, including social distancing, personal hygiene, and acquisition of protective immunity by vaccinations [107].

It is uncertain whether diabetes mellitus alter the immunogenicity of vaccines. An Italian study of 150 participants showed that the presence of diabetes and hyperglycemia did not affect the kinetics, durability, and quantity of neutralizing antibody productions [108]. The protective immunity lasted at least 6 months during the study, with similar titers as those without diabetes [109]. However, this is contradictory to another study of BNT162b2 (Pfizer), which showed that type 2 diabetes reduced vaccine efficacy with an odds ratio of 0.73 (95% CI, 0.59–0.91), compared with those without diabetes [110]. Similar findings were observed in the study of centrally obese patients (define as body mass index higher than 30 kg/m^2^) receiving the mRNA vaccine developed by Moderna: patients with higher waist circumference had significantly lower SARS-CoV-2 antibody titers (*p* = 0.004) [111]. In terms of safety, no serious adverse events requiring hospitalizations were reported. Adiposity parameters such as higher waist circumference, waist-to-hip ratio, body-mass index, or body fat were not associated with more adverse events [111].

In spite of the uncertainty, diabetic patients were first recommended to receive Oxford–AstraZeneca (ChAdOx1-S recombinant) in the early days by the WHO. However, it was found to be unsafe due to the increased risk of venous thromboembolism related to induced antibodies to platelet factor 4 (Anti-PF4) [21,112,113].

More recently, the American Diabetic Association and Centre for Disease Control and prevention advocated prioritizing vaccination to diabetic patients in order to minimize their infection risks [114,115].

### 2.8. Cancer

Cancer patients are at high risk of COVID-19 severe infections due to their age, disease, cancer treatment, and medical co-morbidities [116]. These patients also receive chemotherapy, immunotherapy, or a combination of anti-cancer treatments, predisposing them to fatal outcomes of COVID-19 infection [117,118]. This is especially the case for patients with hematological malignancies, lung cancer, and active malignancy of solid organs [119].

COVD-19 vaccination in cancer patients is safe. An Israeli study involving 170 cancer patients receiving immune checkpoint inhibitors showed that there were no additional safety issues for cancer patients receiving vaccinations [120]. Incidence of systemic serious events was comparable to general healthy populations, including fatigue (4%), headache (3%), myalgia (2%), and chills (1%) [120]. A shorter duration of the second dose, such as 21 days, is recommended to maximize adequate immune response, as shown in an interim study of BNT162B2 (Pfizer) trials of cancer patients [86]. The second dose should not be delayed since one dose of the BNT162b2 (Pfizer) vaccine yields poor efficacy [121]. Possible underlying reasons are poorly understood. It was postulated that some anti-cancer therapy modalities may induce myelosuppression to both myeloid and lymphoid lineages, thus weakening the vaccine immunogenicity and reactogenicity [122,123]. In view of the global vaccine shortage [124], these patients should be prioritized for vaccinations within 21 days.

Several cancer organizations recommend early vaccination if there are no contraindications, due to the possible fatal consequence of severe COVID-19 infections [125,126,127]. In patients with hematological malignancy, it is likely that the benefits outweigh the risks because most cancer treatments nowadays should not prevent the generation of active immunity [128]. Refinement of anti-cancer regimen, close monitoring of medical co-morbidities, and optimization of function status may be considered prior to cancer patients receiving the vaccination. A switch to immunotherapy is a viable choice since it has been shown to have an acceptable safety profile during the vaccination period [120].

### 2.9. Pregnancy

Pregnant patients infected with COVID-19 are at a higher risk for preterm birth and pregnancy loss, ranging from 10% to 25%, and even up to 60% in women with critical illness [129]. They are also at a higher risk of intensive care unit admission, mechanical ventilation, and death [130]. Vaccination during pregnancy is common to prevent maternal and infant morbidity from other infectious diseases, such as influenza and pertussis [131]. Likewise, COVID-19 vaccination aims to reduce the susceptibility to COVID-19 infections to provide a safe pregnancy environment, given the safety and efficacy profiles of the vaccines.

In terms of immunogenicity, a recent study of mRNA COVID-19 vaccine (developed by Moderna) in pregnant and lactating women showed a promising antibody response in infant cord blood and breast milk [132]. Binding and neutralizing antibody titers against the SARS-CoV-2 B.1.1.7 and B.1.351 variants were, however, reduced. This was similarly recorded in another study involving 131 vaccine recipients, showing that mRNA vaccine (Moderna) generated robust immunity in pregnant and lactating women, similar to that of non-pregnant women [133]. Immune transfer of antibodies to neonates also occurred via placenta and breastmilk, particularly for patients receiving vaccinations during their third trimester [134]. However, the preliminary findings of mRNA COVID-19 vaccines (Moderna) involving 35,691 pregnant women did not show an obviously safe profile of vaccinations [135]. A total of 13.9% of recipients resulted in a pregnancy loss, and 81.6% resulted in a live birth (mostly among participants with vaccination in the third trimester). Adverse neonatal outcomes were also reported: preterm birth (9.4%) and small size for gestational age (3.2%), while no neonatal death was reported. Further study with a specific focus on receiving vaccinations in the third trimester is required to maximize the benefits while reducing the risks to pregnancy.

Vaccination of BNT162b2 (Pfizer) in lactating women showed robust SARS-CoV-2-specific IgA and IgG antibodies in breast milk 6 weeks after initiation [136]. Antibodies found in breast milk showed strong neutralizing effects, which may suggest a potential protective effect against infection in the infant. No serious adverse events were documented. Common self-limiting adverse events after the second dose were: local injection-site pain (40.5%), fatigue (33.3%), and fever (11.9%). Another study also showed consistent conclusions that the elevated IgA and IgG antibodies resulted in a lower infant respiratory illness after maternal vaccination of BNT162b2 (Pfizer) [137]. This was further validated in an Israeli study involving 1094 participants from eight hospitals, showing a successful transfer of immunoglobulins to the fetus across the placenta [138]. This led to a substantial rise of anti-SARS-CoV-2 antibody titer in the neonatal blood within 14 days of the first vaccine dose [138]. Vaccinations in very early pregnancy (earlier than 4 months) may not be optimal because the continuous cytotrophoblast layer between syncytiotrophoblast and stromal cells prevents penetration of the villi by IgG [139]. The timing of vaccination in pregnancy should thus be adjusted to at least after the fourth month of pregnancy to maximize active transport of IgG, thus providing protective immunity to both maternal and fetal sides.

In view of the uncertainty of the impact of COVID-19 vaccinations in pregnant women, there have been opposing opinions as to whether pregnant women should receive the vaccination. The American College of Obstetricians and Gynecologists (ACOG), American Society for Reproductive Medicine (ARSM), and the Society for Maternal–Fetal Medicine (SMFM) advocate vaccination in all pregnant and lactating women [140,141,142], while the World Health Organization (WHO) advocates vaccinations only in high-risk pregnant women such as medical care workers or those with co-morbidities that add to the risk of severe diseases [143].

## 3. Contraindications of Vaccination

Several contraindications have been listed by guidelines and pharmaceutical companies. Absolute contraindications are listed in Table 1. The United States Centre for Disease Control and Prevention recommends absolute contraindications in two scenarios [144]:

1. History of a severe allergic reaction (e.g., anaphylaxis) after a previous dose or to a component of the COVID-19 vaccine.
2. Immediate allergic reaction of any severity to a previous dose or known (diagnosed) allergy to a component of the COVID-19 vaccine.

The components of the COVID-19 vaccine are listed in Table 2.

Patients with absolute contraindications should reassess their risk of vaccination and refer to an allergy immunologist. A longer observation period (e.g., 30 min) after vaccination is recommended if they have an immediate allergic reaction or minor contraindications [144]. They may also choose to receive alternative COVID-19 vaccination from other brands without their allergic components. Currently, 24 COVID-19 vaccines have been granted emergency use authorizations by national regulatory authorities (as at 13 September 2021). The first seven vaccines listed in Table 2 have been approved for emergency or full use by at least one WHO-recognized stringent regulatory authority (Pfizer, Moderna, Janssen, Sinovac, Oxford–AstraZeneca, Serum Institute of India Covishield, Sinopharm-BBIBP).

To our knowledge, relative contraindications are overwhelmingly exhaustive because of different situations. However, representative contraindications and recommended actions are listed in Table 3. Subjects with relative contraindications are recommended to discuss individual risk profiles to plan their vaccination decision. Counseling should include risk factors, relative contraindications, benefits and risks of vaccinations, alternative vaccines, and risks of continuing without vaccinations.

## 4. Conclusions

Vaccination, in general, is effective in protecting high-risk populations against severe COVID-19 infections and COVID-19-associated mortality. A summary of special population groups with regards to their features, prognosis of infection, and vaccination decision based on current evidence is listed in Table 4. Patients without contraindications should be prioritized for vaccination under the careful supervision of healthcare workers after balancing the benefits and risks of vaccinations. Adjustment of current medications and treatments may be required in some circumstances, in accordance with guidelines and recently published data. Healthcare workers should optimize medical co-morbidities, closely monitor the post-vaccination disease control and side effects, and seek multidisciplinary collaborations in difficult management of adverse events or interactions with baseline diseases. More research of COVID-19 vaccination in special populations in the future for a thorough understanding of the vaccination outcomes in special populations should be performed. Public education is required to correct patients’ misbelief for a higher vaccination rate globally.

## Figures and Tables

**Table 1:** Absolute contraindications of vaccinations.

Absolute Contraindications	Type of Vaccine	Recommended Actions
Severe allergic reaction, e.g., anaphylaxis	All [144]	1. Do not vaccinate 2. Referral to allergy immunologist 3. Consider other vaccine alternatives
Immediate allergic reaction	All [144]	1. Risk assessment 2. Referral to allergy immunologist 3. Prolong observation period after vaccination (e.g., 30 min)

**Table 2:** Components of 24 COVID-19 vaccines with emergency use authorizations by national regulatory authorities (as at 13 September 2021). The first 7 vaccines on the table have been approved for emergency or full use by at least one WHO-recognized stringent regulatory authority (Pfizer, Moderna, Janssen, Sinovac, Oxford–AstraZeneca, Serum Institute of India Covishield, Sinopharm-BBIBP). The remaining vaccine candidates were arranged in alphabetical order.

Type of Vaccine	Active Ingredient	Inactive Ingredients
Pfizer (mRNA) [145] The United States	Nucleoside-modified mRNA encoding the viral spike (S) glycoprotein of SARS-CoV-2	2-polyethylene glycol (PEG)-2000-N, N-ditetradecyclacetamide cholesterol 1,2-distearoyl-sn-glycero-3-phosphocholine (4-hydroxybutyl) azanediyl)bis(hexane-6,1-diyl)bis(2-hexyldecanoate) sodium chloride monobasic potassium phosphate potassium chloride dibasic sodium phosphate dihydrate sucrose
Moderna (mRNA) [146] The United States	Nucleoside-modified mRNA encoding the viral spike (S) glycoprotein of SARS-CoV-2	PEG2000-DMG: 1,2-dimyristoyl-rac-glycerol,methoxypolyethylene glycol 1,2-distearoyl-sn-glycero-3-phosphocholine cholesterol SM102: heptadecane-9-yl 8-((2-hydroxyethyl)(6-oxo-6(undecyloxyl)hexyl)amino) octanoate tromethamine tromethamine hydrochloride acetic acid sodium acetate sucrose
Janssen (viral vector) [147] The United States	Recombinant, replication-incompetent Ad26 vector encoding a stabilized variant of the SARS-CoV-2 spike (S) protein	polysorbate-80 2-hydroxypropyl-beta-cyclodextrin citric acid monohydrate trisodium citrate dihydrate sodium chloride ethanol
Sinovac/Coronavac (Vero cell) [148] China	Inactivated SARS-CoV-2 virus (CZ02 strain)	aluminum hydroxide disodium hydrogen dodecahydrate sodium dihydrogen phosphate monohydrate sodium chloride
Oxford–AstraZeneca Vaxzevria [149] The United Kingdom	Chimpanzee adenovirus encoding the SARS-CoV-2 Spike (S) protein ChAdOx1-S	L-histidine L-histidine hydrochloride monohydrate magnesium chloride hexahydrate polysorbate 80 (E 433) sucrose disodium edetate (dihydrate)
Serum Institute of India Covishield (Oxford–AstraZeneca formulation) [46,150] India	Recombinant, replication-deficient chimpanzee adenovirus vector encoding the SARS-CoV-2 Spike (S) protein in genetically modified human embryonic kidney 293 cells	L-histidine L-histidine hydrochloride monohydrate magnesium chloride hexahydrate polysorbate 80 (E 433) sucrose ethanol sodium chloride disodium edetate dihydrate (EDTA)
Sinopharm-BBIBP (inactivated virus in Vero cells) [151] China	Inactivated SARS-CoV-2 virus (HB02 strain) in Vero cell culture	aluminum hydroxide adjuvant beta-propiolactone disodium hydrogen phosphate sodium dihydrogen phosphate sodium chloride
Sputnik V (viral vector) [152] Russia	Modified replication-deficient Ad26 and Ad5 encoding the SARS-CoV-2 spike(S) protein	tris-(hydroxymethyl)-aminomethane sodium chloride sucrose magnesium chloride hexahydrate disodium EDTA dihydrate polysorbate 80 ethanol
Abdala [153,154,155] Cuba	Protein subunit vaccine containing COVID-19-derived proteins	No clinical results and information on ingredients found on electronic databases (PubMed, Google Scholar, Medline, Scopus, Embase)
Chinese Academy of Medical Sciences Covidful [156,157] China	Inactivated virus vaccine	No clinical results and information on ingredients found on electronic databases (PubMed, Google Scholar, Medline, Scopus, Embase)
Cansino Convidecia [158,159] China	Recombinant replication-deficient adenovirustype 5-vectored vaccine expressing full-length spike gene based on Wuhan-Hu-1 (Genebank accession number YP_009724390)	Details of inactive components were not listed
Covaxin [160,161], India	Whole-virion inactivated SARS-CoV-2 antigen (strain: NIV-2020770)	aluminum hydroxide imidazoquinolinone 2-phenoxyethonol phosphate-buffered saline
COVIran Barakat [162,163] Iran	Inactivated SARS-CoV-2 virus with Vero cell culture	aluminum hydroxide modified egg’s medium fetal bovine serum
CoviVac [164,165] Russia	Inactivated SARS-CoV-2 virus (strain:AYDAR-1) with Vero cell culture	beta-propiolactone aluminum hydroxide disodium phosphate dihydrate sodium dihydrogen phosphate dihydrate sodium chloride
EpiVacCorona [166,167] Russia	Chemically synthesized peptides (short fragments of viral spike protein) conjugating to a carrier protein containing nucleocapsid proteins and maltose-binding proteins	L-histidine aluminum hydroxide
FAKHRAVAC [168,169] Iran	Inactivated SARS-CoV-2 virus based with cell culture	Details of ingredients not published
Medigen [170,171,172] Taiwan	Recombinant S-2P spike protein adjuvanted with CpG 1018	CpG 1018 aluminum hydroxide phosphate buffer solution
Minhai [173,174,175] China	Inactivated SARS-CoV-2 virus based with Vero cell culture	Details of ingredients not published
QazCovid-in [176,177] Kazakhstan	Inactivated SARS-CoV-2 virus based with cell culture	Details of ingredients not published
Sinopharm-WIBP [178,179,180] China	Inactivated SARS-CoV-2 virus (strain WIV-04) in Vero cell culture	aluminum hydroxide disodium hydrogen phosphate sodium dihydrogen phosphate sodium chloride
Soberana [181,182,183] Cuba	Receptor binding domain of SARS-CoV-2 spike protein conjugated chemically to tetanus toxoid	Details of ingredients not published
Sputnik light [184,185] Russia	Recombinant replication-deficient Ad26 encoding the SARS-CoV-2 spike(S) protein	tris-(hydroxymethyl)-aminomethane sodium chloride sucrose magnesium chloride hexahydrate disodium EDTA dihydrate polysorbate 80 ethanol
Zifivax [186,187] China	Recombinant tandem repeat dimeric receptor-binding domain-based protein subunit vaccine	aluminum hydroxide Details of ingredients not published
ZyCoV-D [188,189] (DNA plasmid vactor) India	DNA plasmid vector carrying the gene encoding the spike protein (S) of the SARS-CoV-2 virus	Details of ingredients not published

**Table 3:** Common relative contraindications of vaccinations reported in the literature and in guidelines. NACI: National Advisory Committee on Immunization; USCDC: United States Centre for Disease Control and Prevention; VITT: vaccine-induced immune thrombotic thrombocytopenia.

Relative Contraindications	Type of Vaccine	Recommended Actions
Acute PCR-confirmed COVID-19 infection	All	Delay vaccination schedule until recovered from acute illness and the criteria for ending isolation have been met [190].
With fever more than 38.5 degrees Celsius	All	Postpone vaccination until fever subsided [190].
High thrombosis and thrombocytopenia risk	AstraZeneca/ COVISHIELD and Janssen	Cautious for patients with history of heparin-induced thrombocytopenia, antiphospholipid syndrome, or major venous or arterial thrombosis with thrombocytopenia after viral vector COVID-19 vaccine [191,192].
Capillary leak syndrome (CLS)	AstraZeneca COVISHIELD	Patients with history of CLS should not receive AstraZeneca vaccine. Vaccination with alternative vaccine is recommended [193].
Myocarditis and pericarditis	Pfizer and Moderna	Defer the second dose schedule if patients developed myocarditis or pericarditis after the first dose. Choice of alternative vaccine or continue with mRNA vaccine should be discussed with medical workers (cardiologist if possible) [96,193].
Pregnancy, planning for pregnancy or breastfeeding	Viral vector vaccines	USCDC recommends safe administration of viral vector vaccine in all trimesters of pregnancy and breastfeeding (as of 11 August 2021) [194]. Canadian NACI recommends viral vector vaccines should be avoided in pregnancy due to elevated risk of VITT [193]. Vaccination is safe during breastfeeding [193].

**Table 4:** Summary table for vaccination decision making in different population groups.

Population Group.	Features	Prognosis after SARS-CoV-2 Infection	Vaccination Decision	References
Adolescents and children	Strong immune preparedness with production of natural antibody (IgM) with broad reactogenicity	Milder symptoms and with better prognosis	Encouraged but based on individual conditions	[24,25,26,27,28,29,30,31,32,33,34,35,36]
Geriatric population	Reduced immunity and age-related organ functional decline	High risk of COVID-19-associaed mortality	To be evaluated based on individual conditions	[37,38,39,40,41,42,43,44,45,46,47,48,49,50,51,52,53,54,55,56,57]
Immunocompromised subjects	Patients with transplant recipients, HIV-infection carriers, and advanced chronic liver diseases are vulnerable to infection	The protective effect and duration of the vaccines are lower in solid organ transplant recipients	Transplant recipients are recommended to receive vaccinations; patients who have just received solid organ transplantation should also delay their vaccination schedule due to induction phase high-dose immunosuppression treatment	[58,59,60,61,62,63,64,65,66,67,68]
Autoimmune diseases	Patients require special care prior to vaccinations	With poor prognosis	Based on risk stratifications of patients and adjustment of timing for vaccinations	[69,70,71,72,73,74,75,76,77,78,79,80,81,82,83,84]
Cardiovascular diseases	Elevated troponin with negative viral serologies; cardiac magnetic resonance imaging (MRI) demonstrated edema and delayed gadolinium enhancement of the left ventricle in a midmyocardial and epicardial distribution	The association between myocardial infarction (MI) and COVID-19 vaccination is unclear	Encouraged but based on individual conditions; deferred vaccination if the disease symptoms are poorly controlled	[85,86,87,88,89,90,91,92,93,94,95,96,97]
Chronic respiratory diseases	SARS-CoV-2 itself causes severe respiratory failure syndrome.	Chronic lung conditions significantly increase the odds of poor clinical outcomes in patients with COVID-19	Patients with respiratory conditions, such as chronic obstructive pulmonary disease, bronchiectasis, cystic fibrosis, and interstitial lung disease, are recommended for vaccination	[98,99,100]
Diabetic and centrally obese populations	Patients with diabetes have a high risk of severe diseases	Increased mortality with COVID-19 infections	Diabetic patients were first recommended to receive Oxford–AstraZeneca (ChAdOx1-S recombinant) in the early days of the pandemic by the WHO	[101,102,103,104,105,106,107,108,109,110,111,112,113,114]
Cancer	Cancer patients are at high risk of severe COVID-19 infections due to their age, disease, cancer treatment, and medical co-morbidities	Patients receiving chemotherapy, immunotherapy, or combination of anti-cancer treatments lead to fatal outcomes of COVID-19 infection	Benefits outweigh the risks; thorough clinical assessment should be performed before vaccination	[115,116,117,118,119,120,121,122,123,124,125,126,127]
Pregnancy	Vaccination during pregnancy is common to prevent maternal and infant morbidity from other infectious diseases, such as influenza and pertussis	COVID-19 pregnant patients are at a risk for preterm birth and pregnancy loss	In view of the uncertainty of the impact of COVID-19 vaccinations in pregnant women, there has been opposing opinions to whether pregnant women should receive vaccination	[128,129,130,131,132,133,134,135,136,137,138,139,140,141,142]

## Data Availability

All data are accessible on electronic databases (PubMed, Google Scholar, Scopus, Embase, Medline, Web of Science).
